# Genetics architecture of spontaneous coronary artery dissection in an Italian cohort

**DOI:** 10.3389/fcvm.2024.1486273

**Published:** 2024-11-25

**Authors:** Marta Casula, Daniela Marchetti, Lucia Trevisan, Laura Pezzoli, Matteo Bellini, Serena Patrone, Antonio Zingarelli, Fabio Gotta, Maria Iascone, Paola Mandich

**Affiliations:** ^1^ Department of Neuroscience, Rehabilitation, Ophthalmology, Genetics, Maternal and Child Health (DINOGMI), University of Genoa, Genoa, Italy; ^2^ Laboratory of Medical Genetics, ASST Papa Giovanni XXIII, Bergamo, Italy; ^3^ Medical Genetics Unit, IRCCS Ospedale Policlinico San Martino, Genoa, Italy; ^4^ Cardiological Unit, Ospedale Policlinico IRCSS San Martino, Genoa, Italy

**Keywords:** whole exome sequencing (WES), *DROSHA*, connective tissue disorder, miRNA, spontaneous coronary artery dissection (SCAD), Fragile X premutation

## Abstract

Spontaneous coronary artery dissection (SCAD) is a relevant non-atherosclerotic cause of acute coronary syndrome with a complex genetic architecture. Recent discoveries have highlighted the potential role of miRNAs and protein-coding genes involved in the processing of small RNAs in the pathogenesis of SCAD. Furthermore, there may be a connection between SCAD and the increased cardiovascular risk observed in fragile X premutation carriers as well as a correlation with pathogenetic variants in genes encoding for collagen and extracellular matrix, which are related to connective tissue disorders (CTDs). In our cohort of 15 Italian SCAD patients, a total of 37 rare variants were identified in 34 genes using whole exome sequencing (WES) and TRIO-WES analysis when both parents were available. Three likely pathogenic/pathogenetic variants were found in genes previously associated with SCAD and CTDs (*COL3A1, COL1A2*, and *SMAD3*) and 26 variants of uncertain significance in genes previously associated with SCAD and CTDs. TRIO-WES analysis revealed 7 *de novo* variants, 1 of which was found in a potential novel candidate gene (*DROSHA*). In addition, a premutation allele of 55 ± 2 CGG repeats in the promoter of the *FMR1* gene was identified in two related SCAD patients by test for CGG-repeat expansions in the 5′-UTR of the *FMR1* gene. Our findings suggest various potential mechanisms such as mRNA toxicity, miRNA regulation, alteration of collagen, and the extracellular matrix architecture, all of which could disrupt vascular homeostasis, and finally, WES and TRIO-WES have proven to be the most powerful approaches for characterizing the genetic background of SCAD.

## 1 Introduction

Spontaneous coronary artery dissection (SCAD) is a non-atherosclerotic cause of acute coronary syndrome (ACS) with a prevalence of 1%–4% (1, 2). It is one of the major causes of acute myocardial infarction in young to middle-aged women (3). Several different risk factors have been associated with SCAD including female sex, pregnancy-related factors, inflammation, emotional and/or physical stressors (4), hypothyroidism (5), migraine (6–8), and hypertension. Another frequently associated condition is fibromuscular dysplasia (FMD) which has been described, in some case series, in more than 50% of the patients (9–11). Although the complex genetic component of SCAD is still unexplained, increasing evidence suggests a multifactorial and polygenic basis with common genetic loci (about 16 associated with SCAD risk have been identified) contributing in the determination of the risk of SCAD (12). Furthermore, a growing number of reports associate SCAD with heritable connective tissue disorders (CTDs) such as Marfan syndrome, vascular Ehlers–Danlos syndrome (vEDs), and Loeys–Dietz syndrome (LDS) (13–17), suggesting an underestimated monogenic rare disease in SCAD (18). Moreover, the role of miRNAs and of genes encoded for proteins involved in processing of small RNAs is rapidly emerging, both in vascular biology and in possible pathogenic mechanisms of vascular diseases (19, 20) and a relationship between toxicity due to Fragile X premutation and the pathogenesis of SCAD has been hypothesized (21). Recently, the association of connective tissue involvement in females with intermediate or gray zone alleles was reported in a cohort of females with CTDs (22).

### 1.1 Aims of the research

Although genome-wide association studies (GWAS) have revealed several loci associated with increased risk of SCAD, the genetic causes of SCAD remain mostly unknown. For this purpose, we collected a homogeneous cohort of Italian patients, phenotypically well characterized and hospitalized for SCAD. This cohort underwent whole exome sequencing (WES) and, when both parents were available, TRIO-WES analysis. The goal of this study was to contribute to elucidate the role of genetic factors in pathological mechanisms underlying SCAD disease and the diagnostic route through genetic testing and clinical genetic evaluation.

## 2 Patients and methods

### 2.1 Study population

Data of SCAD patients evaluated at IRCCS Policlinico San Martino in Genoa between January 2010 and July 2023 were collected retrospectively. The inclusion criterion was SCAD diagnosis formulated in the catheterization laboratory. Fifteen patients out of 14 families meeting this criterion were enrolled. After the hospitalization for SCAD event and complete recovery, a genetic evaluation was offered to all patients. Clinical, therapeutic, electrocardiographic, echocardiographic, and angiographic data were gathered and analyzed. All patients underwent a regular clinical and instrumental follow-up (at 1, 6, and 12 months after SCAD and then every year, unless otherwise indicated), during which non-invasive imaging by coronary computed tomography angiography (CCTA) was employed to evaluate the spontaneous healing of the involved coronary vessels. Moreover, FMD was assessed by different imaging techniques. A detailed genetic visit was performed for all patients and their available relatives. A complete personal and three-generation family history was collected with a particular focus on cardiovascular disease and CTDs (see Family tree in the Supplementary Material)*.* All patients and their parents underwent a specific physical evaluation for CTDs, including Beighton scale and Marfan scores. During genetic counseling, limits and implications of the study were discussed, and a signed informed written consent was collected. The local Ethical Committee approved the study (22 May 2022, n. 858).

### 2.2 Genetic analyses

Genomic DNA of patients and available parents was extracted from peripheral blood using commercial DNA extraction kits. WES analysis was performed at the Molecular Genetic Laboratory of ASST-Papa Giovanni XXIII of Bergamo.

The exonic and flanking splice junction regions were captured using the Clinical Research Exome v.2 kits (Agilent Technologies, Santa Clara, CA, USA). Sequencing was performed on a NovaSeq6000 Illumina system with 150 bp paired-end reads. Reads were aligned to human genome build GRCh38, and the minimum coverage of the samples was 10×. The variant call file, including single nucleotide polymorphism and indels, was annotated by querying population frequencies databases and mutation databases, including the Genome Aggregation Database (http://gnomad.broad institute.org/), ClinVar (https://www.ncbi.nlm.nih.gov/clinvar/), and Human Gene Mutation Database Professional (HGMD, Release 2023.3). To prioritize variants, a sequential filtering strategy was applied, retaining only variants with the following characteristics: (a) potential effect on protein and transcript (splicing, missense, nonsense, and frameshift); (b) consistency with the patient's phenotype according to a selected panel of genes (23) previously associated with CTD and vasculopathies or previously identified in SCAD patients (23); (c) consistency with the suspected inheritance model (X-linked, autosomal recessive or *de novo*) if parents were available. Variants were classified based on current guidelines (24, 25). The potential causative variants were subsequently confirmed by Sanger sequencing in the proband and parents. As previously described, two pipelines were used to identify the copy number variants (CNVs) based on ExomeDepth (26, 27). All patients were analyzed for CGG-repeat expansions in the 5′-UTR of the Fragile X messenger ribonucleoprotein 1 (*FMR1*) gene according to the manufacturer's instruction (FRAXA analysis, AmplidexTM kit, Asuragen, Austin, TX, USA) and evaluated following current guidelines (28, 29).

## 3 Results

### 3.1 Population characteristics

The cohort of 15 SCAD cases comprised 14 (93%) female Italian patients of European ancestry with an average age at the SCAD event of 46 years (range: 30–58 years). Two women in the cohort were sisters.

The clinical characteristics of SCAD patients are summarized in Table 1. Among all SCAD, two (13%) were pregnancy-related SCAD (P-SCAD), defined as SCAD occurring during pregnancy or within 12 months of delivery, and only one patient (6.7%) suffered a recurrence SCAD episode. Migraine was present in eight (53.3%) patients.

**Table 1:** SCAD patient characteristics.

Features	Study cohort
Female, *n* (%)	14 (93)
Age at first SCAD event, mean (range)	46.1 (30–58)
SCAD recurrence, *n* (%)	1 (6.7)
Pregnancy-related SCAD, *n* (% female)	2 (13)
Current smoker, *n* (%)	3 (20)
Past smoker, *n* (%)	3 (20)
Hypertension, *n* (%)	5 (33.3)
Hypercholesterolemia, *n* (%)	4 (26.6)
Anxiety–depressive disorder, *n* (%)	5 (33.3)
Migraine, *n* (%)	8 (53.3)
Hypothyroidism, *n* (%)	3 (20)
Stress event, *n* (%)	3 (20)

Stressor factors at the time of SCAD event were present in three (20%) patients and five (33.3%) had anxiety–depressive disorders. The most common cardiovascular risk factor was hypertension (33.3%). The clinical presentation of SCAD was chest pain in all patients, but in three (20%) patients, the clinical onset was complicated by cardiac arrest. The tortuosity of the coronary vessels with a diameter ≥2 mm, defined as the presence of ≥3 consecutive curvatures of 90°–180° evaluated at end-diastole (30), was found in five patients (33.3%) during coronary angiography. Most patients 10 (66.6%) had a preserved anterograde coronary flow with initial thrombolysis in myocardial infarction (TIMI) 3. A percutaneous coronary intervention (PCI) was performed in three (20%) patients with initial absent antegrade flow (TIMI 0), ongoing chest pain, and persistent ST elevation on electrocardiogram. A conservative management with antiplatelet therapy was the prevalent strategy (80%) as suggested in recent ACS guidelines (31) proving to be the most effective as reported in some studies, in which a conservative approach is effective in the most part of patients (32–34) (Supplementary Table S1).

Clinical genetic evaluation excluded the presence of CTDs diagnosis in the probands, but two patients (13.3%) showed asymptomatic vascular abnormalities on imaging: one had a dissection of both internal carotid arteries and the other one had dissection of one of the anterior branches of the duplicated right vertebral artery. FMD was absent in our cohort. Beighton scale and Marfan scores were ≤2 in all patients.

### 3.2 Genetic results

Fifteen SCAD patients underwent WES analysis, first using a focused approach on SCAD-associated genes previously identified by different genetic approaches. All cases have read depth ≥10× for ≥98% of the consensus coding sequence of analyzed genes, suggesting adequate coverage to detect protein-coding single nucleotide variants and indels. TRIO-WES analysis was performed in five trios.

We found 37 rare variants in 34 genes. We found three likely pathogenic/pathogenetic (LP/P) variants in genes previously associated with SCAD and CTDs (*COL3A1*, *COL1A2*, and *SMAD3*) (Table 2).

**Table 2:** Likely pathogenic and pathogenic variants (ACMG classification) in CTDs and SCAD-related genes.

Case ID	Gene	Nucleotide variant	Amino acid variant	Family study	GnomAD v4.1 MAF^a^
2	COL1A2	NM_000089.4: c.670C>T	p.(Arg224Cys)	NA	0.0000018590
4	SMAD3	NM_005902.4: c.28C>G	p.(Pro10Ala)	NA	6.215 × 10^−7^
7	COL3A1	NM_000090.4: c.117T>G	p.(Tyr39ter)	*De novo*	0

NA, not available.

^a^ GnomAD MAF refers to all populations.

Twenty-six variants were classified as variants of uncertain significance (VUS) in genes both previously associated with SCAD and CTDs or already described (30–33) (Supplementary Table S2). We found three variants in genes candidate for SCAD but also described in association with arterial dissection/aneurism (*TLN1*, *SMAD6*, and *TSR1*) (35–38).

Patient carrying the LP missense variant c.28C > G; p.(Pro10Ala) in *SMAD3* gene associated with LDS type 3 showed coronary arteries tortuosity and scoliosis since puberty. During the follow-up, a progressive increase in the aortic root diameter was observed.

Patient with LP variant c.670C > T; p.(Arg224Cys) in *COL1A2* present no involvement of other vascular districts and no signs of CTDs and/or osteogenesis imperfecta.

By TRIO-WES analysis, despite the small cohort of patients, we got seven *de novo* variants, one in a possible novel candidate gene (*DROSHA*), and four in genes not apparently correlated with SCAD or CTDs (*SHANK2*, *SUPV3L1*, *SBNO1*, and *MCHR2*) (Supplementary Table S3).

One variant [c.2277C > A; p.(Ser759Arg)] was found in *COL4A1.* The patient carrying this variant did not have any symptoms or signs associated with the COL4A1 syndrome.

A *de novo* truncating heterozygous LP/P variant was found in *COL3A1* c.117T > G p.(Tyr39Ter), not previously reported in literature and absent in general population databases.

This variant was found in a patient with an asymptomatic dissection of both internal carotid arteries diagnosed after P-SCAD event.

The only male patient of the cohort had the *DROSHA* c.2317G > A p.(Val773Ile) variant. He presented a SCAD event at 45 years worsened by a cardiac arrest. He did not show any sign of CTDs and had a positive familial history for aortic aneurism (paternal uncle at 70 years) and P-SCAD (paternal cousin before 35 years).

The only two related SCAD patients of our cohort are two sisters who carried a premutation allele of 55 ± 2 CGG repeats in the promoter of *FMR1* gene (Supplementary Table S4). One sister had a SCAD event at 37 years and the other one at 45 years. Both had subsequent multiple events of chest pain without new ACS events, but they did not present any involvement of other vascular districts nor any sign of CTDs.

## 4 Discussion

SCAD is no longer considered a rare condition. However, its true prevalence remains unknown largely due to underdiagnosis.

According to previous studies, we suggest three different mechanisms that could contribute to the production of this complex phenotype.

### 4.1 mRNA toxicity and miRNA regulation

The increased risk of cardiovascular disease in Fragile X premutation carriers is already well defined. Recently, SCAD has been reported to be associated with Fragile X premutation (22, 39). These reports suggest a possible role of the Fragile X premutation in SCAD pathogenesis through different mechanisms. First, Fragile X Messenger Ribonucleoprotein (FMRP) mild deficiency influences extracellular matrix (ECM) degradation contributing to vasal dissection. Its diminished level leads to abnormal connective tissue structure including shortened and fragmented elastin fibers (21). Moreover, the *FMR1* premutation could affect the component of the elastin matrix in connective tissue. Second, the fragile X premutation causes an elevation of the amount of mRNA transcript, which is known to be linked to mRNA toxicity through the increase of RNA binding proteins bound to RNA. Among these, the sequestration of DROSHA/DGCR8 has the effect of downregulating miRNAs, essential for maintaining cellular normal functions (40–42). Endothelial cells may be more sensitive to miRNA dysregulation than other tissues (43). Furthermore, women with premutation may have many medical issues related to their condition, such as hormone replacement therapy for ovarian insufficiency (FXPOI), hypertension, and psychological stress, which overlap with known risk factors for SCAD (35, 44, 45).

**Figure 1:**
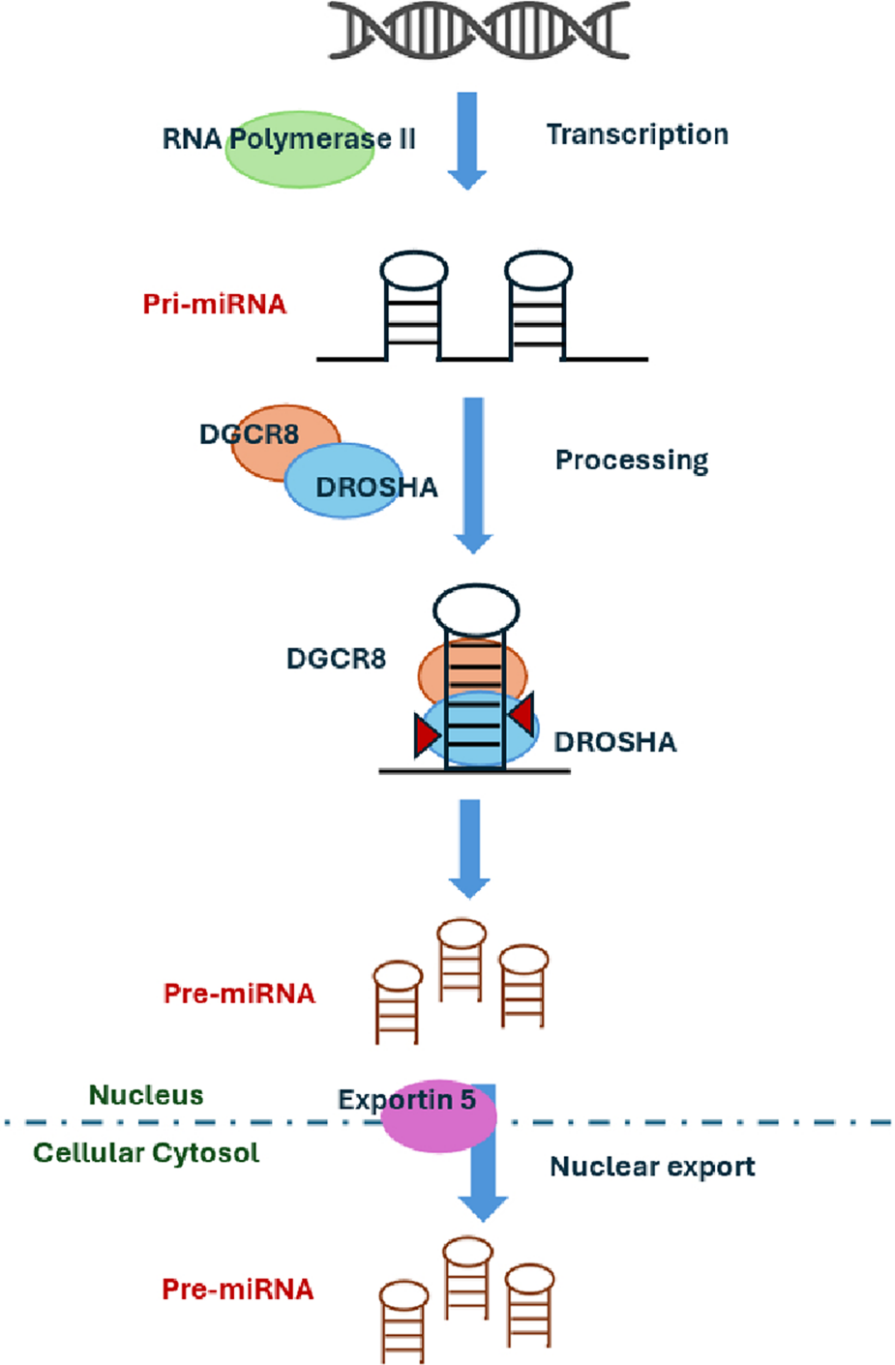
The role of DROSHA in miRNA production.

In our study, we identified two sisters with *FMR1* premutation at the lower limit of the range. The definition of the number of repeats associated with disease manifestations is traditionally related to intellectual disability. Historically, the limit between intermediate allele and premutation *FMR1* allele was defined considering the risk of expansion to full mutation. Novel interest in other clinical aspects such as cardiovascular involvement is now emerging, and the border of these intervals could be reevaluated when these aspects will be taken into consideration. The role of the premutation *FMR1* allele in the severity of our patient's clinical picture deserves further investigations. Recently, four candidate miRNAs have been identified as promising biomarkers for acute SCAD: miR-let-7f-5p, miR-146a-5p, miR-151a-3p, and miR-223-5p. These miRNAs were significantly expressed in the cohort of SCAD patients. The target genes of these four miRNAs are associated with vascular biology and TGF-beta signaling (20). To further support the role of miRNA dysregulation in SCAD pathogenesis, we found a *de novo* variant in *DROSHA* or ribonuclease III nuclear (RNASEN, OMIM*608828), an essential microRNA processing enzyme engaged in the process for maturing microRNA. miRNA biogenesis is initiated by transcription with RNA polymerase II (46) and their primary transcripts (pri-miRNAs) that harbor a local hairpin structure then cropped by a nuclear RNase III, DROSHA, into ∼70 nt precursor-miRNAs (pre-miRNAs). DROSHA works in a microcomplex nature in the nucleus and processes pre-miRNAs into precursor miRNA that contains a dsRNA-binding protein, DGCR8, required for vascular development (Figure 1). It has been shown that DGCR8 binds preferentially to expansion of CGG repeats in the 5′ UTR of *FMR1* gene*,* thus showing a link between miRNAs regulation, Fragile X Syndrome, and vascular development. DROSHA, by processing miRNA, regulates vascular development and homeostasis (41). Therefore, the loss or reduction of ubiquitously expressed DROSHA may predispose to vascular damages. It is demonstrated that DROSHA is essential for fetal and postnatal endothelial development and promotes vascular development and homeostasis in vertebrates (43). This finding suggests the potential involvement of DROSHA in human vascular disorders. Interestingly, missense variants of *DROSHA* mediate primarily vascular abnormalities in humans. Since DROSHA is involved in vascular homeostasis, its role in determining SCAD events should be taken into consideration together with miRNA regulation. If confirmed, this may suggest experimenting treatments with miRNA mimics or antagonists to improve clinical features in patients with vascular abnormalities.

### 4.2 Role of collagen

Robust evidence showed the involvement of 10 collagen genes among the ECM structural constituent: the most common genes with interesting variants were *COL3A1*, *COL5A1*, and *COL4A1* (14, 23, 47, 48).

In our cohort we identified 11 rare variants (18% LP/P) in collagen genes. Pathogenetic variants were found in *COL3A1* and *COL1A2* genes (Table 2).

The role of collagen genes (especially *COL3A1* and *COL5A1*) in arterial dissection and FMD has been described already (47, 48), and spontaneous dissection has been demonstrated in a murine model (47).

*COL3A1* associated with vEDs encodes for pro*α*1chain of type 3 collagen, and it is the most associated with arterial dissections and/or ruptures of variable severity (47, 49). Our patient with a *COL3A1* variant presents only vascular signs. In fact, not only the vEDs but also a less severe phenotypic spectrum has been described in the last few years in association with *COL3A1* variants (50–53).

The *COL1A2* gene, classically linked with osteogenesis imperfecta and Ehler-Danlos syndrome (EDS), has been recently associated with hereditary thoracic aortic aneurysm and dissection disease complex (TAAD), suggesting a genetic overlap between SCAD and TAAD (54).

Moreover, a VUS variant was observed in *COL4A1* gene. *COL4A1* was already described as associated with a wide spectrum of small-vessel brain disease including porencephaly, variably associated with eye defects and systemic findings (kidney involvement, muscle cramps, cerebral aneurysms, Raynaud phenomenon, cardiac arrhythmia, and hemolytic anemia). Our patients did not show any of these signs.

The role of collagen genes is significant even in our smaller cohort (detection rate 2/15, 13%), highlighting the indication to analyze collagen pathways in SCAD patients for the important implications in their surveillance.

Therefore, our findings confirm the role of collagen genes in SCAD pathogenesis even in patients not fulfilling the clinical diagnostic criteria.

### 4.3 Extracellular matrix and role of cell-to-cell interaction

ECM is a complex and dynamic structure involved in the physiological and pathological processes of the cardiovascular system. The cardiomyocytes are interconnected by the ECM to form a multicellular syncytium that allows the coordinated myocardial excitation and contraction. Collagen is the main component of heart ECM and its quantitative/qualitative alterations have effects on myocardial contraction, relaxation, diastolic stiffness, and electrical conduction. ECM composes the vascular structure and provides endothelial functions such as the expression of adhesion molecules, the formation of tight junctions, and the endothelial metabolism. During pregnancy, ECM is an important component of blood vessel remodeling with a potential role in cardiovascular disease, such as P-SCAD. Moreover, the ECM plays a significant role in the inflammatory system, contributing to vascular pathologies, as observed in SCAD (7, 55, 56).

An LP variant in *SMAD3* was observed in one patient in our cohort. The SMAD family proteins are a group of intracellular signal transducer proteins like the gene products of the *Drosophila* gene “mothers against decapentaplegic” (Mad) and the *Caenorhabditis elegans* gene Sma. SMAD3 functions in the transforming growth factor-beta signaling pathway and transmits signals from the cell surface to the nucleus, regulating gene activity and cell proliferation. This protein forms a complex with other SMAD proteins and binds DNA, functioning both as a transcription factor and tumor suppressor. Variants in this gene are associated with aneurysms-osteoarthritis syndrome and LDS3. Our patient with *SMAD3* variant presented, at follow-up, mild signs of LDS, as described above.

Several studies found enrichment in rare variants in *PKD1* gene, both PV and VUS, in SCAD patients with and without kidney involvement (14, 17, 27, 47, 57, 58). *PKD1* encodes a membrane protein, polycystin-1, involved in cell-to-cell or cell-to-matrix interactions. This protein, besides its well-known role in kidney disease, is implicated in the structural integrity of blood vessels with a potential role in SCAD pathogenesis (17): VUS in *PKD1* were found in heterozygous form in 14% of our patients, without any kidney involvement.

*MYH11* is one of the four genes associated with the dysregulation of smooth muscle cells contractile function (*ACTA2*, *MYH11*, *MYLK*, and *PRKG1*) (59, 60).

*MYH11* encodes the smooth muscle–specific myosin heavy chain, and its genetic variants in patients with arterial dissections demonstrate its role in inducing structural vascular fragility due to defects in focal adhesion, intercellular adhesion, and the actomyosin network (61, 62).

One of our patients presented a VUS in *MYH11*. Albeit this finding is intriguing, the role of this gene in SCAD pathogenesis remains to be proven.

*TLN1* is a large gene highly expressed in the vasculature, including aorta, tibial, and coronary arteries. It encodes for a protein engaged in focal adhesion and essential for integrin activation. Alterations in the expression of *TLN1* in smooth muscle cells have been associated with major risk of human arterial diseases, confirming the importance of talin 1 in vascular integrity (63). A study demonstrated that *TLN1* was significantly downregulated in aortic tissue of patients with aortic dissection (64), and another case report showed rare missense variants in *TLN1* associated with familial and sporadic SCAD cases (65). Furthermore, a murine model showed that the inactivation of talin 1 leads to defects in angiogenesis (66). A rare variant of *TLN1* was found in one of our cases with SCAD, dissection of one of the anterior branches of the duplicated right vertebral artery and positive paternal family history. Therefore, our findings support the role of ECM and the need of investigating this group of genes in SCAD (diagnostic rate 1/15, 6.7%).

## 5 Conclusion

In our study, we collected data from a small cohort of SCAD patients and, although this is a limited case series, the detection rate of LP/P variants has been higher than expected and previously reported (20%) (17, 47).

This discrepancy can be due to the small sample of our cohort and the inclusion criteria used; however, our results underline the importance of studying the genetics of these patients for the possible diagnosis of unexpected systemic disorders.

These findings, together with the increasing evidence from recent literature, support the idea that nowadays, accurate genetic diagnosis and multidisciplinary assessment are important to improve the management in SCAD patients.

The characterization of the genetic background could change in the future the complex working diagnosis and could personalize patient care, familial ascertainment, and management and enable access to specific treatments. Although in a limited series, our data showed that, due to the large number of the genes involved in SCAD, WES-TRIO is the most powerful approach to identify variants in genes already associated with SCAD and to discover new pathways that can contribute to elucidate the complex genetic architecture of SCAD.

## Data Availability

The datasets presented in this article are not readily available because due to ethical/privacy restrictions. Italian law does not allow the deposit of exome data in a public repository. Furthermore, patients have not authorized us to deposit genetic data. Requests to access the datasets should be directed to the corresponding author.
