# Supplementary material for: Genetics architecture of spontaneous coronary artery dissection in an Italian cohort
Source: Front Cardiovasc Med. 2024 Nov 25;11:1486273. doi: 10.3389/fcvm.2024.1486273 (PMC11625805; doi:10.3389/fcvm.2024.1486273)
Supplement: Supplementary file 1 [file Datasheet1.pdf]

Supplementary Material

Table S1. Clinical onset, angiographic characteristic and management of SCAD event

**Clinical onset n (%)**	
STEMI	12 (80)
NSTEMI	3 (20)
**Angiographic characteristic n (%)**	
Tortuosity	5 (33,3)
TIMI 0	3 (20)
TIMI 1	1 (6,66)
TIMI 2	1 (6,66)
TIMI 3	10 (66,6)
**Location of SCAD event**	
LAD	11 (73,3)
OM	-
RCA	3 (20)
PDA	3 (20)
RPL	1 (6,66)
D1	-
D2	1 (6,66)
D3	-
LCx	1 (6,66)
**Management n (%)**	
PCI management	3 (20)
Conservative management	12 (80)
**Antiplatelet strategy n (%)**	
SAPT	5 (33,3)
DAPT	10 (66,6)
**Antiplatelet therapy n (%)**	
Cardioaspirin	14 (93,3)
Ticagrelor	4 (26,6)
Clopidogrel	6 (40)
Prasugrel	1 (6,66)

STEMI:ST elevation myocardial infarction
NSTEMI: non ST-segment elevation myocardial infarction
LAD: left anterior descending artery
OM:obtuse marginal
PCI: percutaneous coronary intervention
RCA: right coronary artery
PDA: posterior descending artery
RPL: right posterolateral
D1:1st Diagonal
D2: 2nd Diagonal
D3: 3rd Diagonal
TIMI: Thrombolysis In Myocardial Infarction
SAPT: Single antiplatelet therapy
DAPT: Dual antiplatelet therapy

Table S2. Description of variants of uncertain significance (VUS) in genes both previously associated with SCAD and CTDs identified in our cohort.

Case ID	Gene	Nucleotide variant	Amino acid variant	Family study	Disease inheritance	GnomAD v4.1 MAF
1	ABCC6	NM_001171.6: c.167A>G	p.(His56Arg)	NA	AD/AR	0.00008552
2	EMILIN1	NM_007046.4: c.920C>T	p.(Ala307Val)	NA	AR	0.000003223
2	COL12A1	NM_004370.6: c.5467G>A	p.(Val1823Ile)	NA	AD/AR	0.0002945
2	COL1A2	NM_000089.4: c.670C>T	p.(Arg224Cys)	NA	AD/AR	0.000001859
2	ADAMTS10	NM_001009944.2: c.1910C>T	p.(Ala637Val)	NA	AD	0
3	COL11A2	NM_080680.3: c.889G>C	p.(Gly297Arg)	NA	AD/AR	0.000007735
3	MYH11	NM_002474.3: c.5392C>T	P.(Arg1798Trp)	NA	AD	0.00001983
5	ATP6V0A2	NM_012463.4: c.2175+5del.	p.?	NA	AR	0.000003718
6	ABCC6	NM_0011715.6: c.4015C>T	p.(Arg1339Cys)	Not M⁂	AR	0.00004778
6	PKD1	NM_001009944.3: c.2081C>T	p.(Pro694Leu)	M	AD	0.01971
6	ADAMTSL4	NM_019032.6: c.232C>T	p.(Leu78Phe)	Not M⁂	AR	0.0002163
7	PLOD1	NM_000302.4 c.1172A>G	p.(Asn438Ser)	F	AR	0.0001137
7	COL2A1	NM_001844.5: c. 3659T>C	p.(Met1220Thr)	F	AD	0.000003113
7	FLNB	NM_001457.4: c.2237A>C	p.(Lys746Thr)	F	AD/AR	0.00001549
8-15*	COL5A1	NM_000093.4: c.700T>C	p.(Tyr234His)	M	AD	0.000055669
8-15*	MTHFR	NM_005957.5: c.*2809T>C	P.?	M	AD/AR	0.00001156
8-15*	COL5A2	NM_000393.5: c.4354-21C>T	p.?	F	AD	0.000003099
8	ALDH18A1	NM_002860.4: c.2116A>G	p.(Thr706Ala)	M	AD/AR	0.000001859
9	LTBP2	NM_000428.3: c.848G>T	p.(Ser283Ile)	F	AR	0.000001286
9	COL4A1	NM_001845.6: c.2277C>A	p.(Ser759Arg)	F	AD	0.00001487
9	B3GALT6	NM_080605.4: c.588dup	p.(Arg197AlafsTer246)	F	AR	0.0001448
10	B3GALT6	NM_080605.4: c.220A>C	p.(Ser74Arg)	NA	AR	0
10	PKD1	NM_001009944.2: c.7148G>T	p.(Arg2383Leu)	NA	AD	0
12	FBN2	NM_001999.4: c.8536C>T	p.(Arg2846Cys)	M	AD	0.00001673
12	NOTCH1	NM_017617.5: c.1505A>G	p.(Asn502Ser)	F	AD	0.000003105
15	COL9A3	NM_001853.4: c.1208G>C	p.(Gly403Ala)	NA	AD/AR	0.00001912

NA: not available; AD: autosomal dominant; AR: autosomal recessive; M: maternal, F: paternal

*: sisters. ⁂: for case 6 only maternal DNA sample was available. The test excluded the maternal inheritance but can not distinguish between paternal or de novo origin.

frontiers

Supplementary Material

Table S3. Candidate genes and novel candidate genes

Case ID	Gene	CG / NCG	Nucleotide variant	Amino acid variant	Family study
8	TSR1	**CG**	NM_018128.5: c.1061C>T	p.(Ser354Phe)	M
9	TLN1	**CG**	NM_006289.4: c.5881-7T>C	p.?	F
5	SMAD6	**CG**	NM_005585.5: c.106G>A	p.(Asp36Asn)	F
8	SBNO1	**NCG**	NM_001167856.2:c.641C>T	p.(Ser214Phe)	de novo
8	SHANK2	**NCG**	NM_012309.5: c.5047G>A	p.(Gly1683Ser)	de novo
8	COL14A1	**NCG**	NM_021110.4: c.847C>T	p.(Arg283Cys)	de novo
9	MCHR2	**NCG**	NM_001040179.2: c.642G>T	p.(Leu214Phe)	de novo
11	DROSHA	**NCG**	NM_013253.5:c.2317G>A	p.(Val773Ile)	de novo
12	SUPV3L1	**NCG**	NM_003171.5: c.196C>T	p.(Pro66Ser)	de novo

M: maternal, F: paternal; CG: candidate gene (SCAD associated candidate genes already described in literature); NCG: novel candidate gene (SCAD associated candidate genes identified in our study)

Supplementary Material

Table S4. FRAXA results.

**Case ID**	**Sex**	***CGG repeats at 5'-UTR FMR1 gene**
1	F	19-31
2	F	30-32
3	F	27-32
4	F	25-30
5	F	31-32
6	F	21-33
7	F	33-42
8*	F	32-55
9	F	13-31
10	F	30-31
11	M	30
12	F	31-32
13	F	29-31
14	F	30-31
15*	F	32-55
*Normal alleles (< 45 CGG);Intermediate allele (45-54 CGG);		
Premutation allele (55-200 CGG), Full mutation (> 200 CGG)		
*: sisters		

Family tree

Legend

SCAD

SCAD + ICA dissection

Cerebral aneurysm

Abdominal aortic aneurysm

Thoracic aortic ectasia

Aortic dissection type A (Stanford classification)

SCD

Cavernous angioma

SCAD + anterior branch of VA dissection

SCAD: spontaneous coronary artery dissection
ICA: internal carotid artery
VA: vertebral artery
SCD: sudden cardiac death

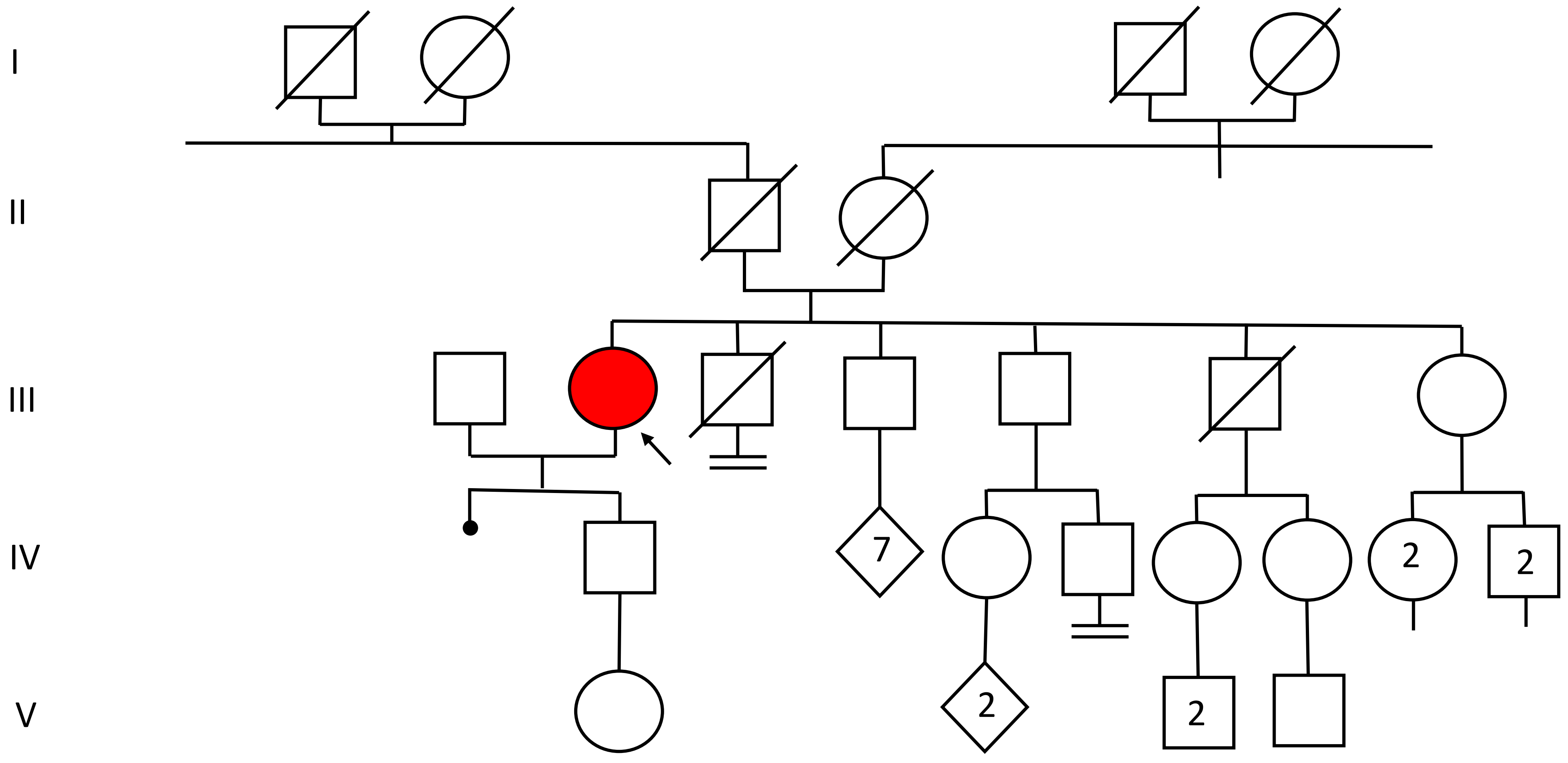

CASE ID 2
COL1A2:p.(Arg224Cys)

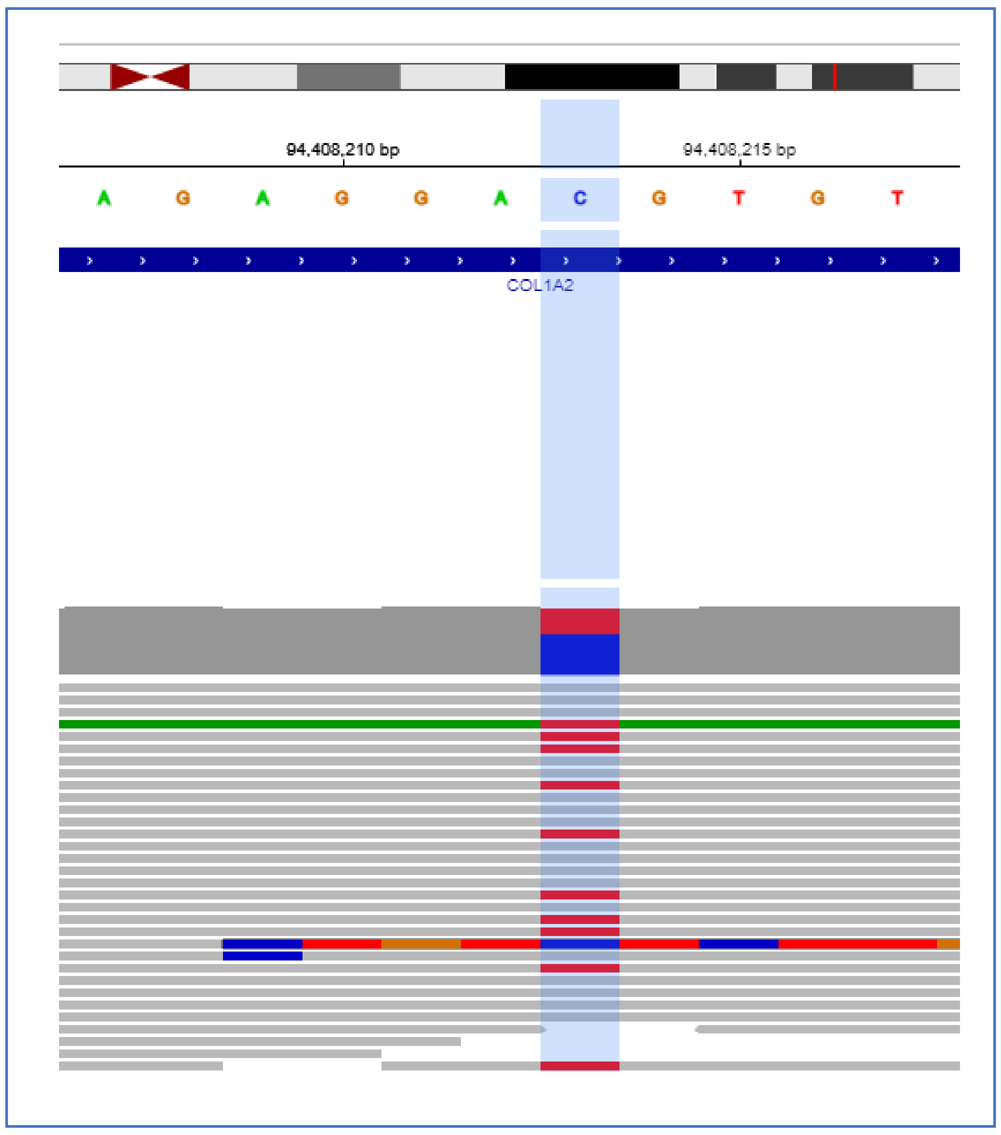

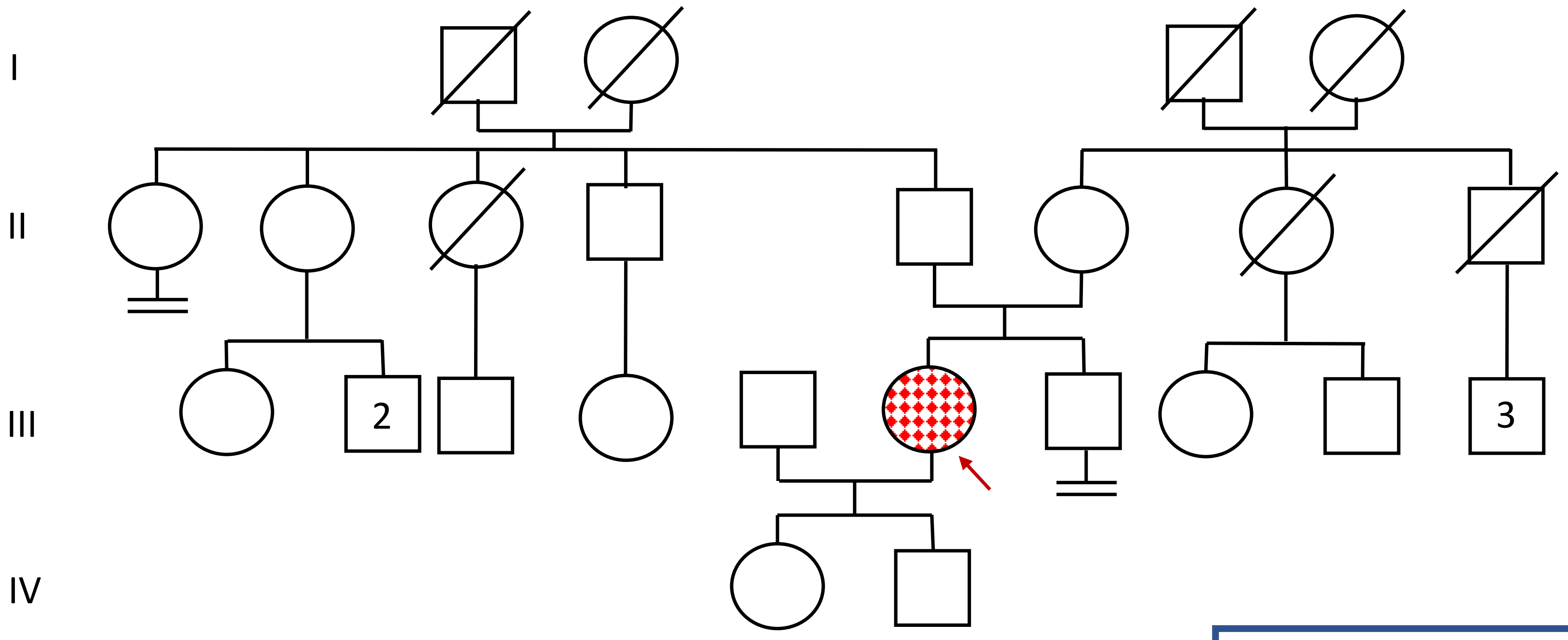

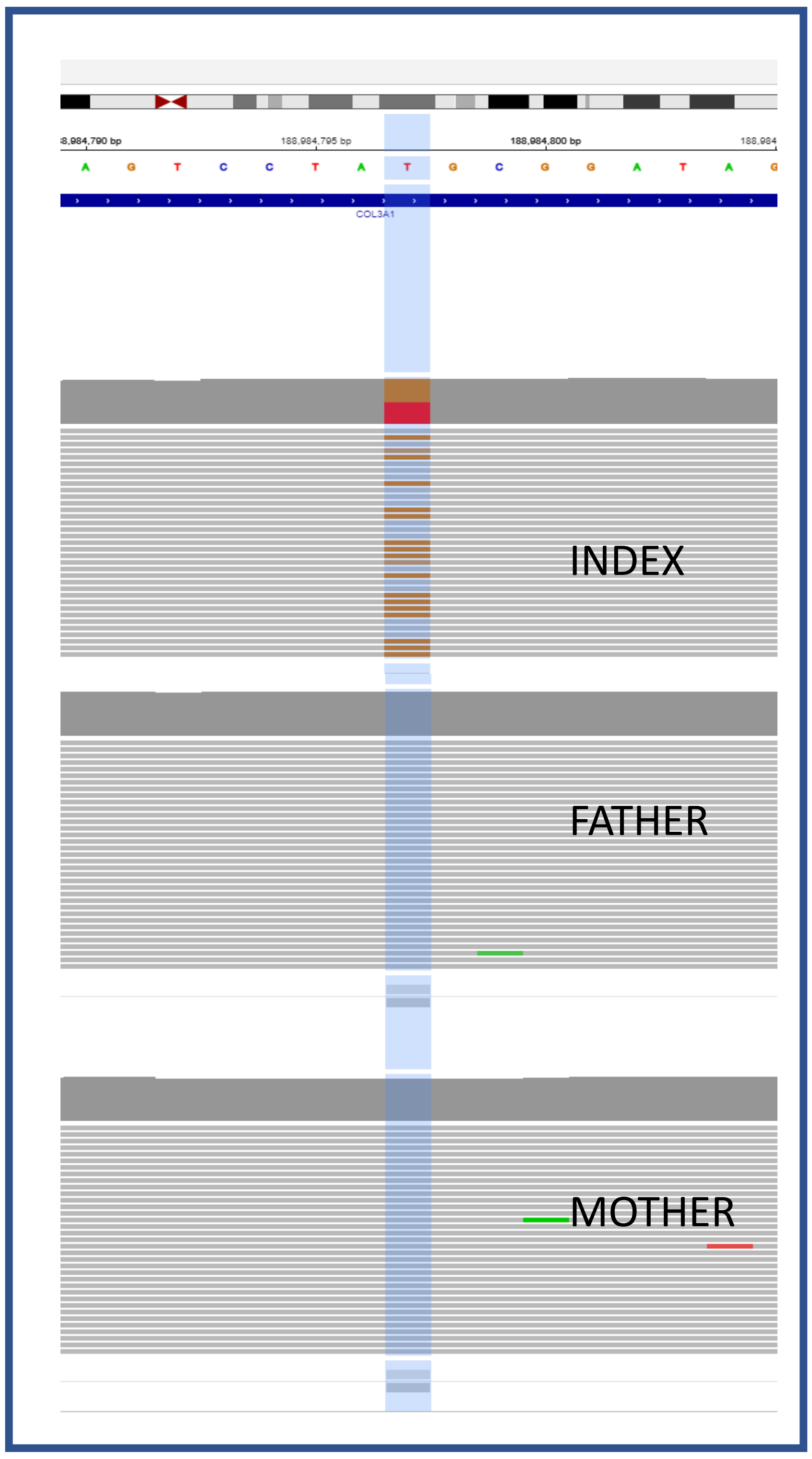

CASE ID 7

COL3A1:p.(Tyr39Ter)
De novo

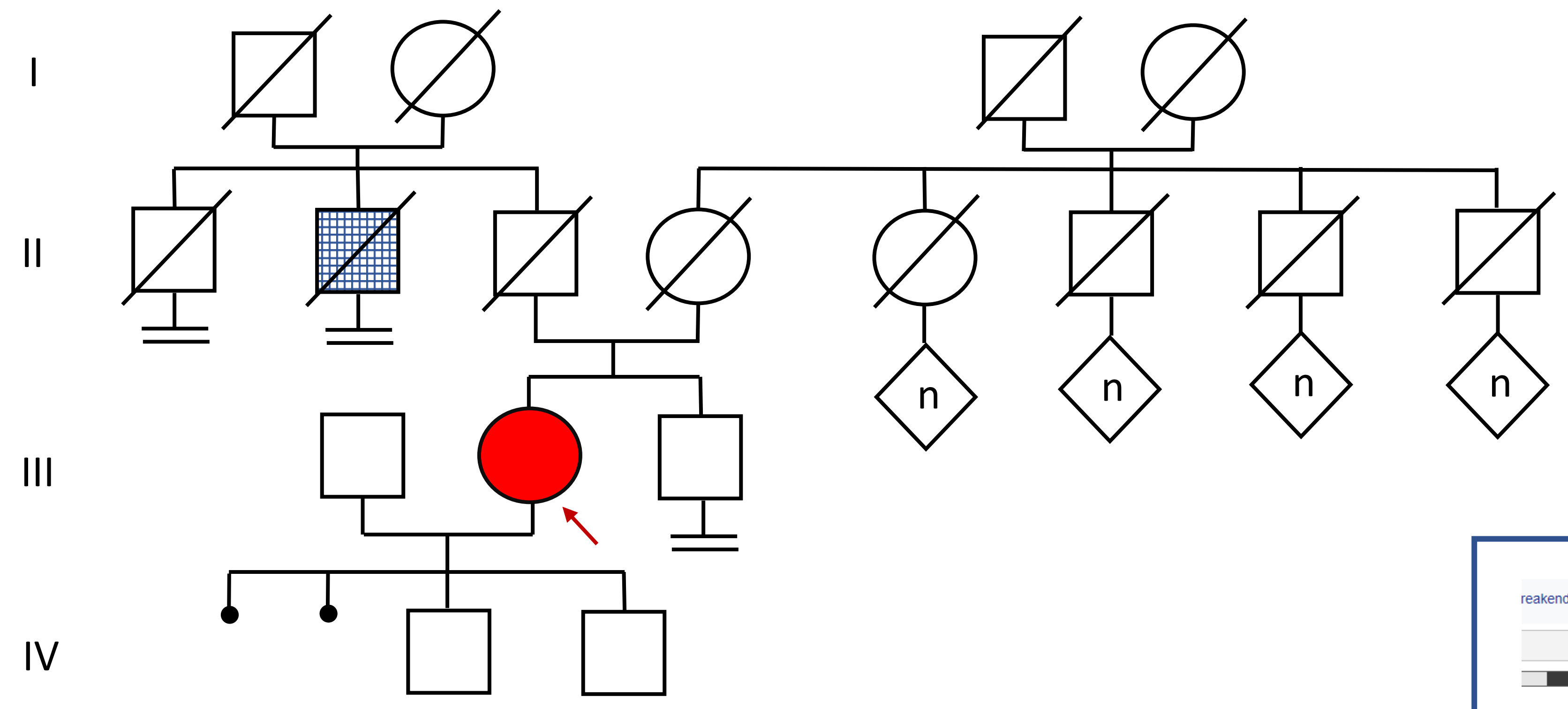

CASE ID 4
SMAD3: p.(Pro10Ala)

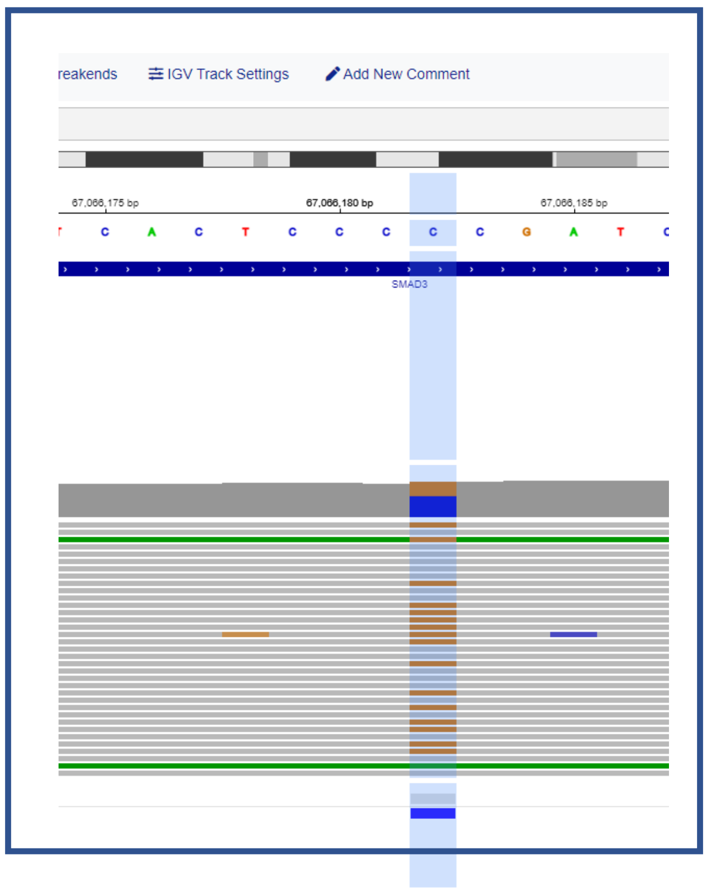

CASE ID 8 AND CASE ID 15

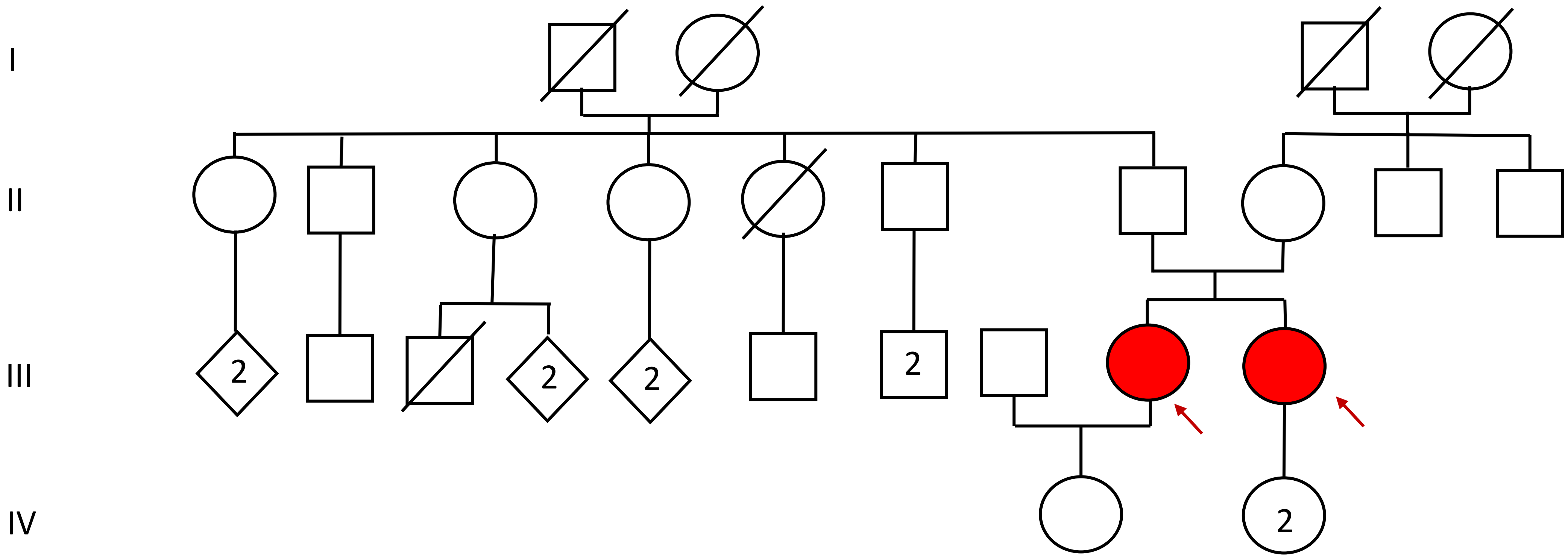

CASE ID 8 (III 10)

COL5A2	c.4354-21C>T	paternal
COL5A1	p.(Tyr234His)	maternal
MTHFR	c.*2809T>C	maternal
TSR1	p.(Ser354Phe)	maternal
ALDH18A1	p.(Thr706Ala)	maternal
COL14A1	p.(Arg283Cys)	de novo
SHANK2	p.(Gly1683Ser)	de novo
SBN01	p.(Ser214Phe)	de novo

CASE ID 15 (III 9)

COL5A2	c.4354-21C>T	paternal
COL5A1	p.(Tyr234His)	maternal
MTHFR	c.*2809T>C	maternal
COL9A3	p.(Gly403Ala)	paternal

CASE ID 12

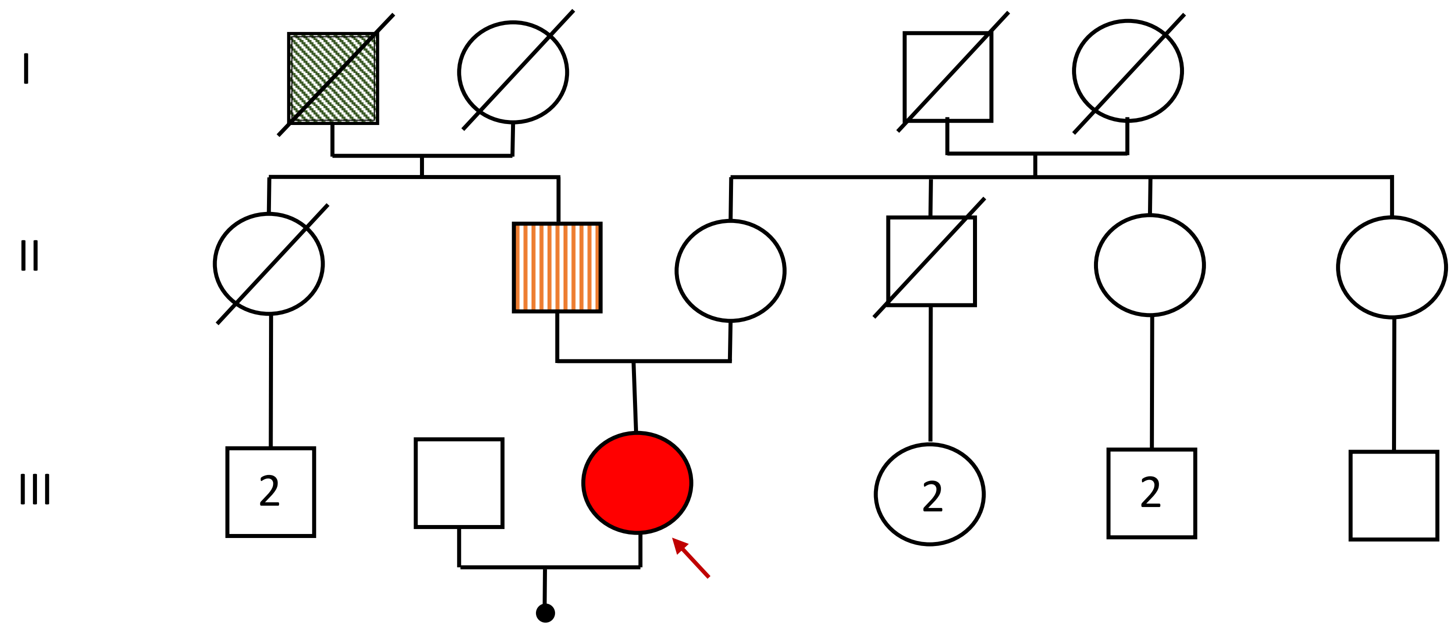

CASE ID 14

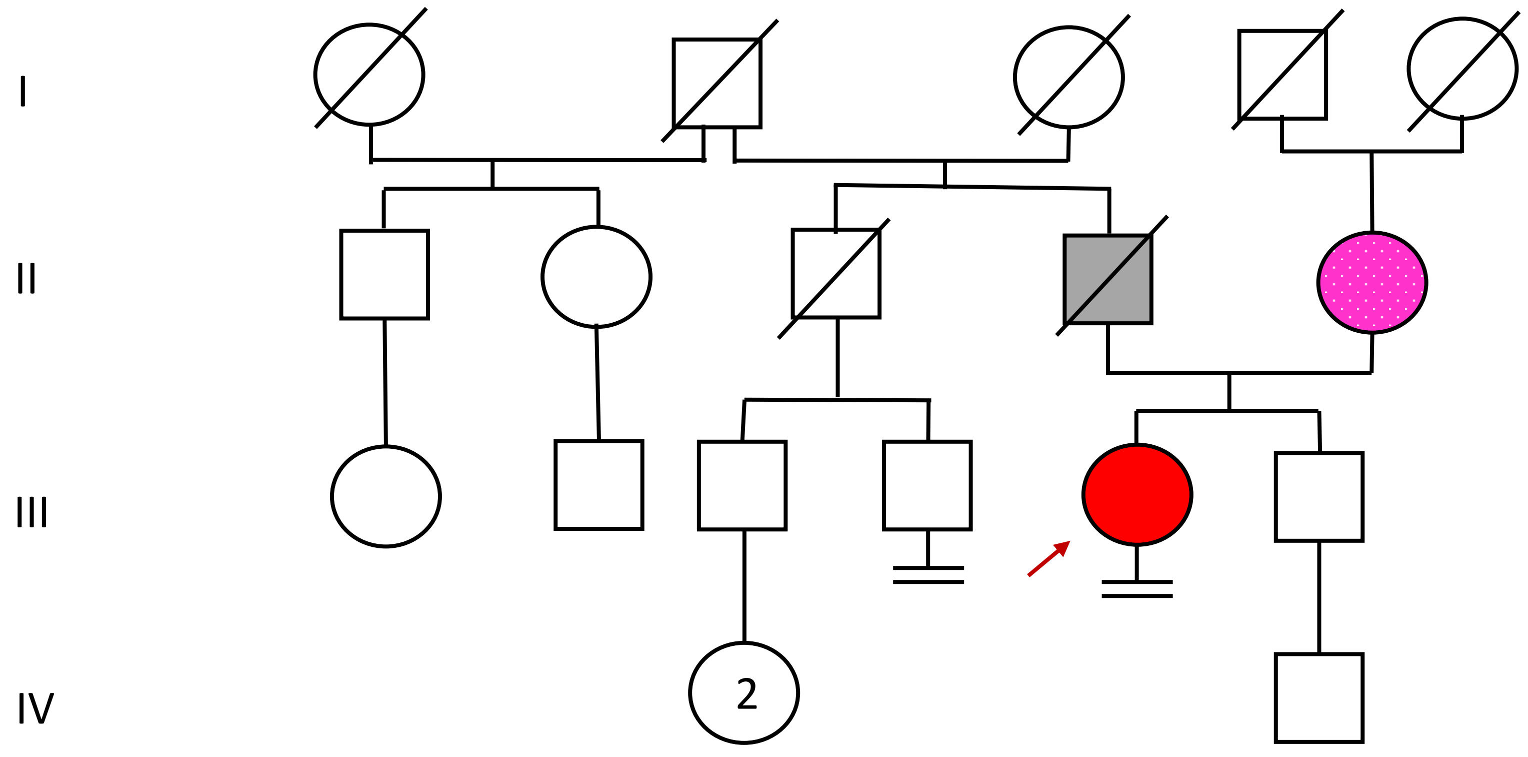

CASE ID 9

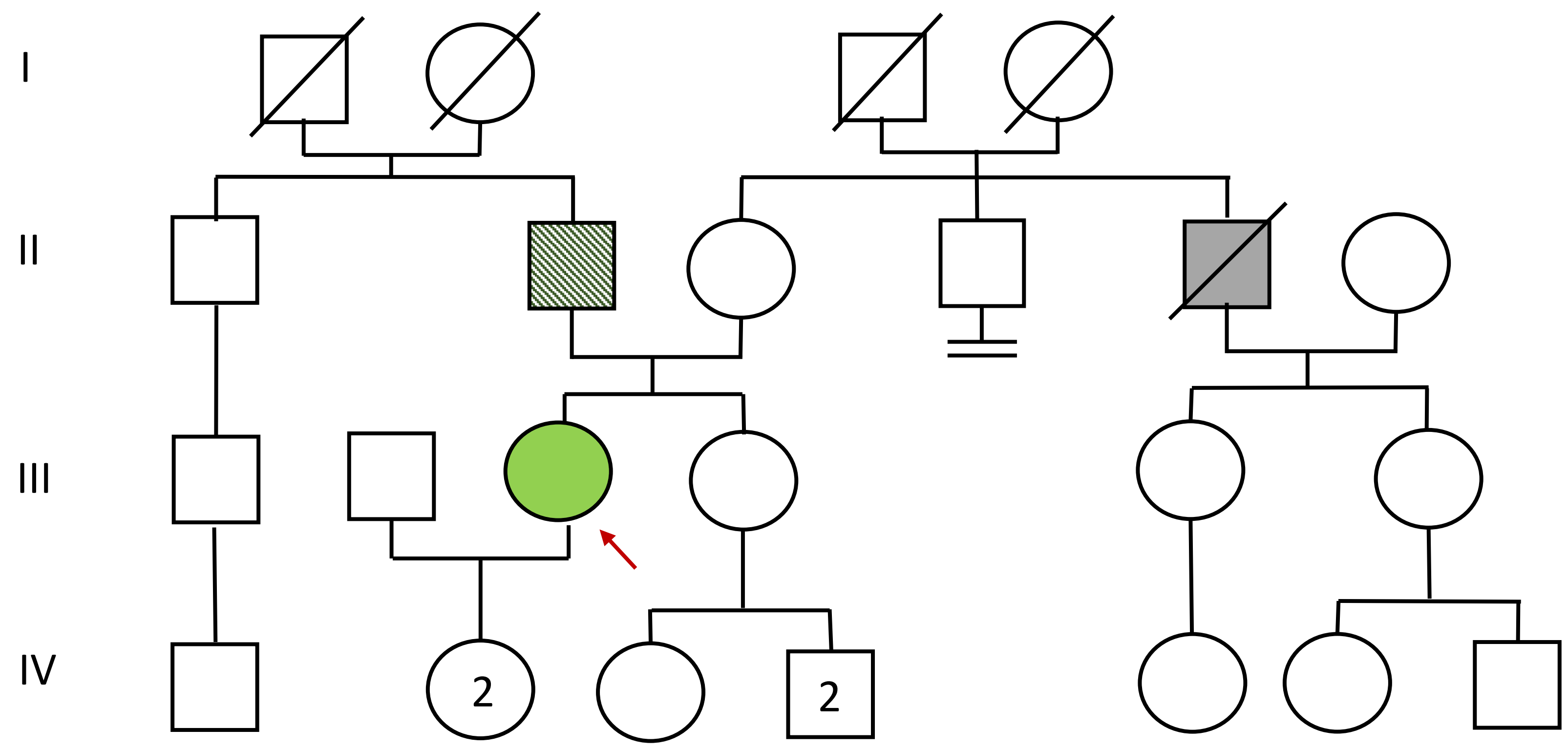

CASE ID 3

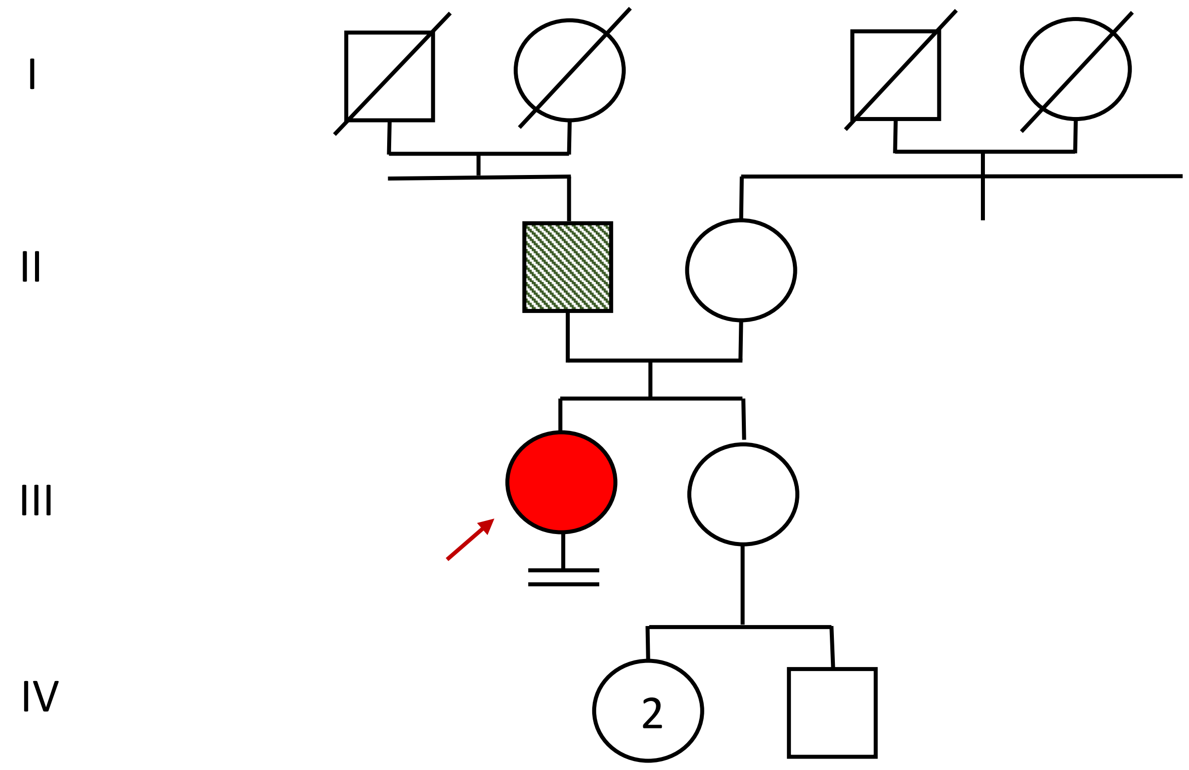

CASE ID 1

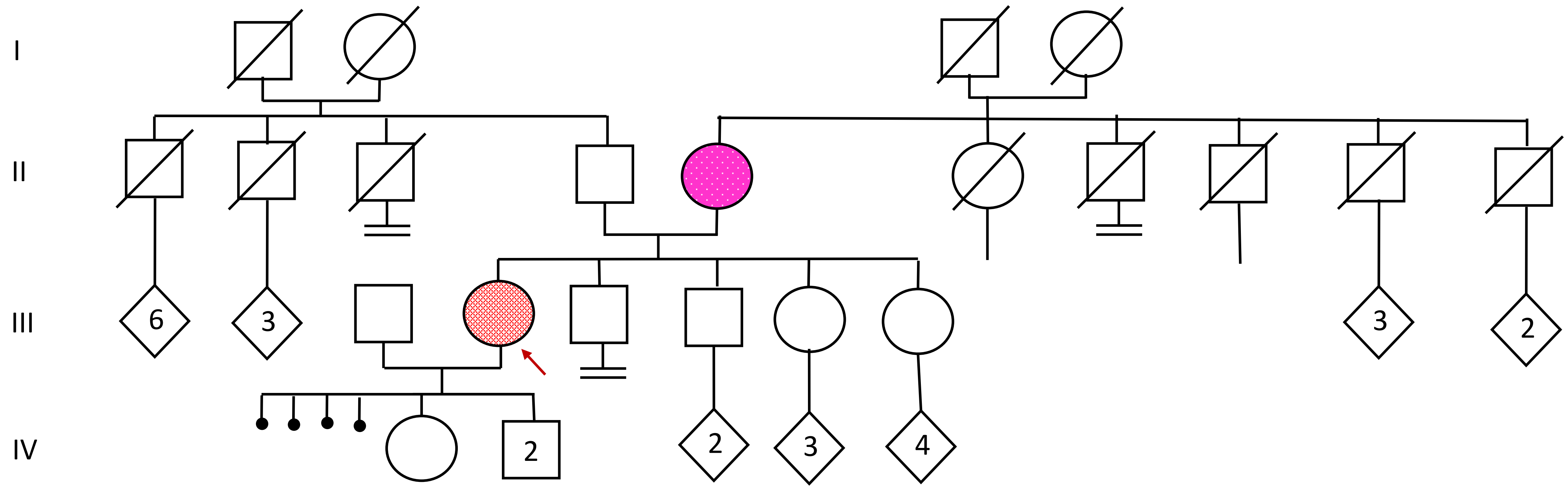

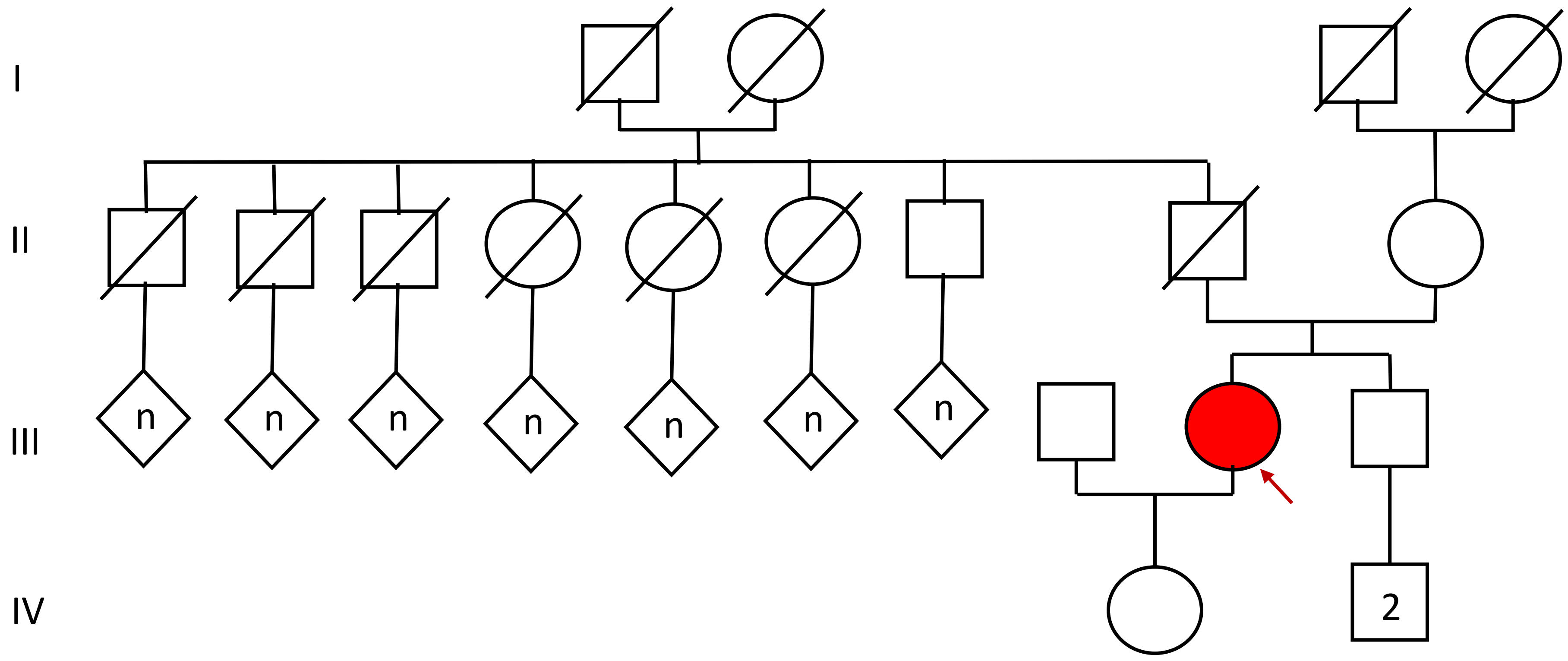

CASE ID 5

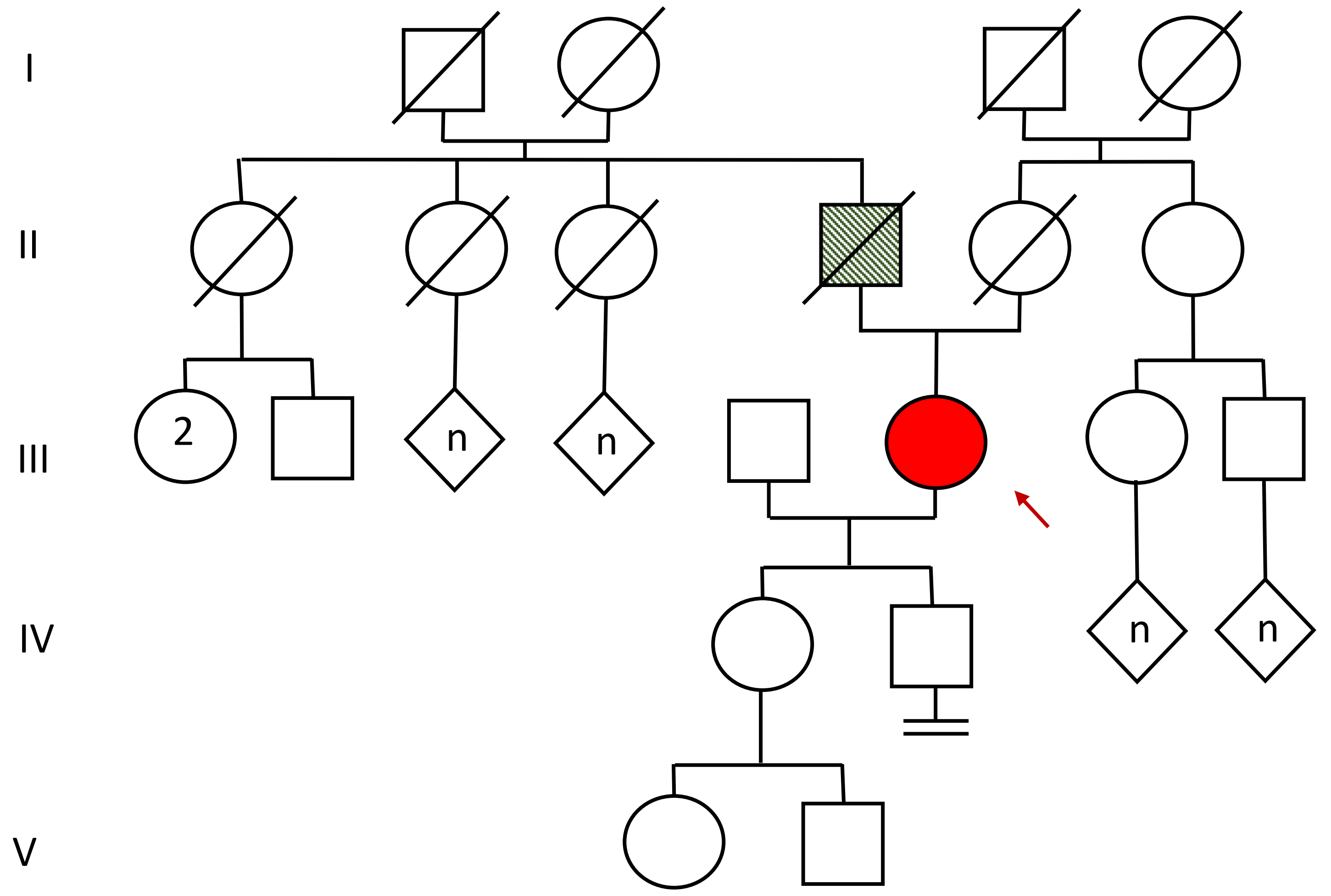

CASE ID 10

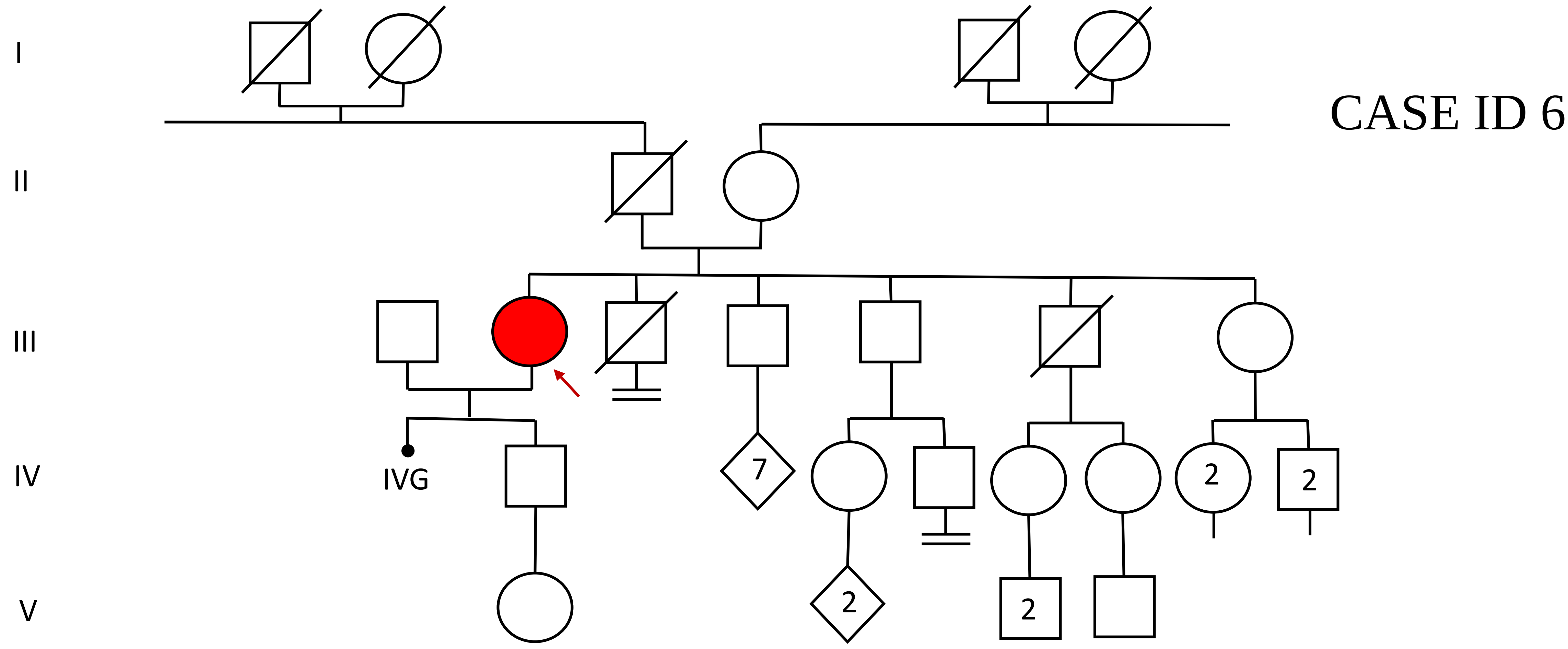

CASE ID 6

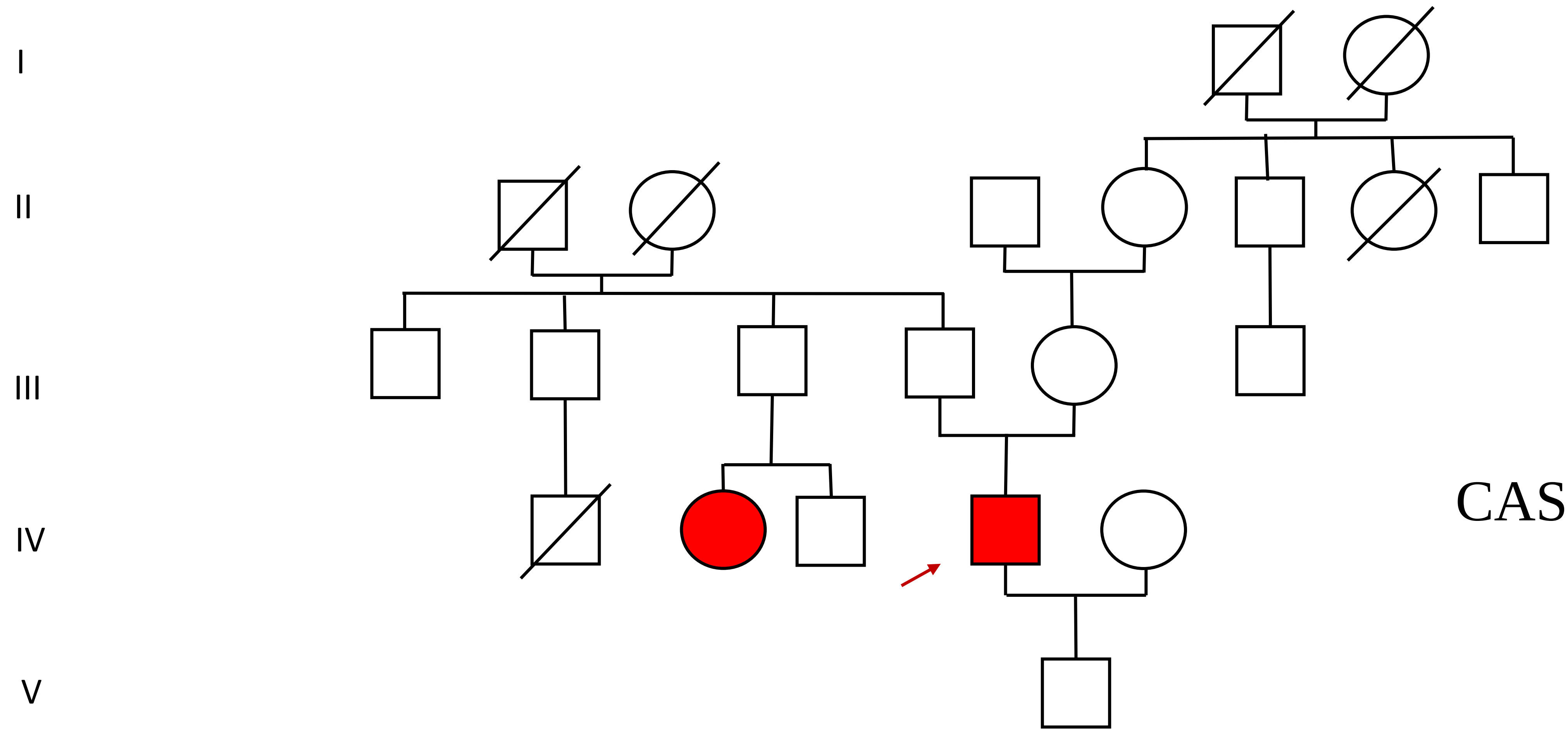

CASE ID 11

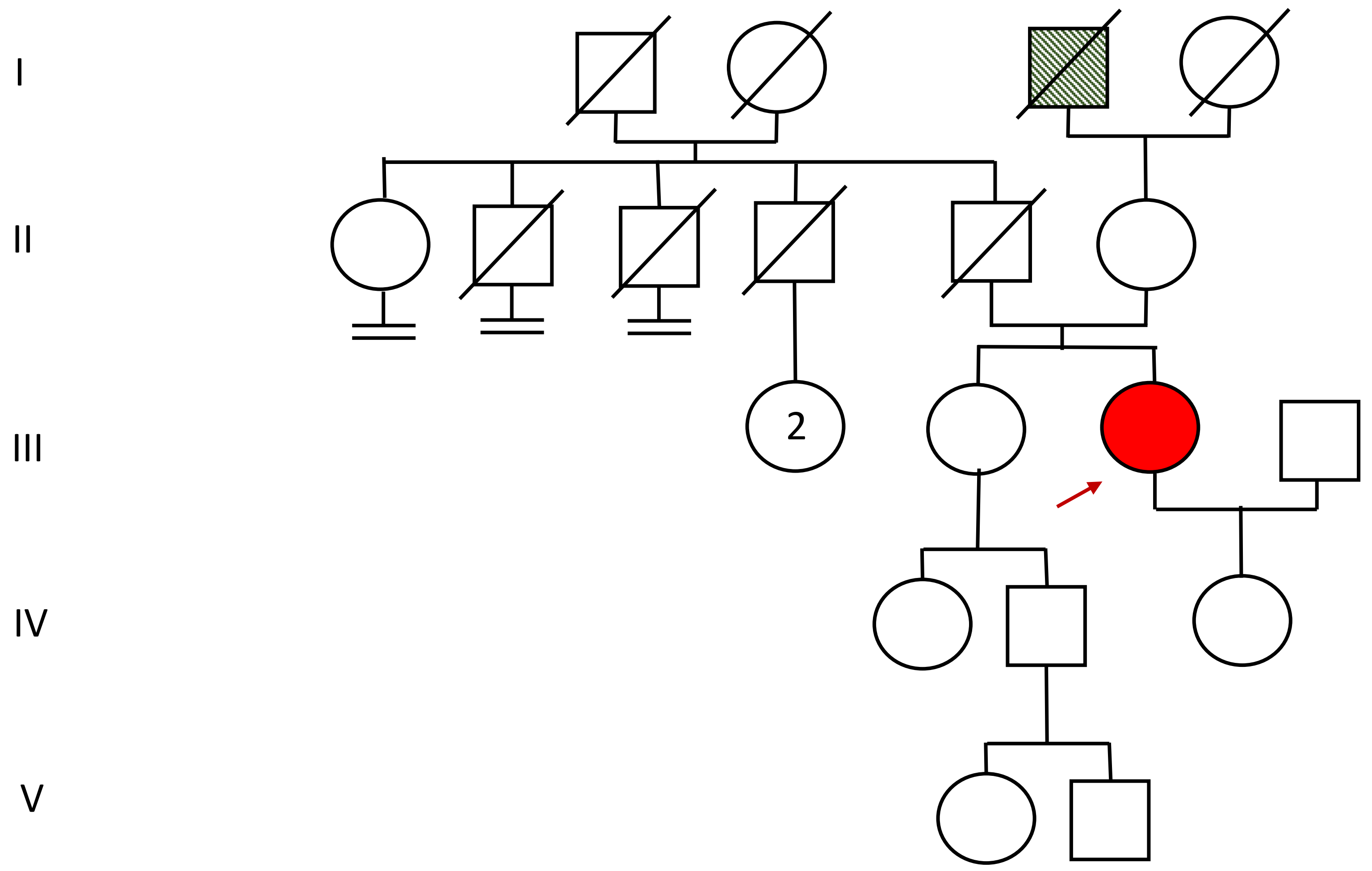

CASE ID 13
